# Supplementary material for: Phylogenomics of white-eyes, a ‘great speciator’, reveals Indonesian archipelago as the center of lineage diversity
Source: eLife. 2020 Dec 22;9:e62765. doi: 10.7554/eLife.62765 (PMC7775107; doi:10.7554/eLife.62765)
Supplement: Supplementary file 1. — Abbreviations for museums: American Museum of Natural History, New York (AMNH); Australian National Wildlife Collection, Canberra (ANWC); Burke Museum of Natural History and Culture, Washington (Burke); Lee Kong Chian Natural History Museum, Singapore (LKCNHM); Museum of Vertebrate Zoology, California (MVZ); Museums Victoria, Melbourne, Australia (NMV); Museum Zoologicum Bogoriense, West Java, Indonesia (MZB); Naturalis Biodiversity Center, Leiden, Netherlands (Naturalis); South Australian Museum, Adelaide (SAMA); Swedish Museum of Natural History, Stockholm (NRM); Western Australian Museum, Perth (WAM); Yale Peabody Museum of Natural History, Connecticut (Peabody). Whole genome resequenced samples are marked with an asterisk (*) at the end of the sample ID. [file elife-62765-supp1.docx]

Supplementary file 1. Details of all samples included in the study. Abbreviations for museums: American Museum of Natural History, New York (AMNH); Australian National Wildlife Collection, Canberra (ANWC); Burke Museum of Natural History and Culture, Washington (Burke); Lee Kong Chian Natural History Museum, Singapore (LKCNHM); Museum of Vertebrate Zoology, California (MVZ); Museums Victoria, Melbourne, Australia (NMV); Museum Zoologicum Bogoriense, West Java, Indonesia (MZB); Naturalis Biodiversity Center, Leiden, Netherlands (Naturalis); South Australian Museum, Adelaide (SAMA); Swedish Museum of Natural History, Stockholm (NRM); Western Australian Museum, Perth (WAM); Yale Peabody Museum of Natural History, Connecticut (Peabody). Whole genome resequenced samples are marked with an asterisk (*) at the end of the sample ID. Raw FASTQ files of target enriched samples are available on GenBank and the assembled loci of all samples are available on Dryad (<https://doi.org/10.5061/dryad.8931zcrmt>).

| Sample ID | Taxon | Sample type | Locality | Coordinates (Lat, Lon) (º) | Date collected | Sources | GenBank BioProject PRJNA682287 | |
| --- | --- | --- | --- | --- | --- | --- | --- | --- |
|  |  |  |  |  |  |  | BioSample no. | SRA |
| AMNH700010 | *Z. simplex* | Toepad | Pulau Pintu, Gedong, Malaysia | - | 1912-Nov-02 | AMNH | SAMN16986199 | SRS7828810 |
| AMNH700035 | *Z. auriventer* | Toepad | Kao Nong, Thailand | - | 1913-Jun-26 | AMNH | SAMN16986202 | SRS7828829 |
| AMNH700016 | *Z. simplex* | Toepad | Sarawak, Borneo, Malaysia | - | 1892-Aug-03 | AMNH | SAMN16986214 | SRS7828813 |
| WAMF1A23240 | *Z. unicus* | Toepad | Flores Island, Indonesia | -8.5833, 120.5 | 1990-May-05 | WAM | SAMN16986200 | SRS7828806 |
| WAMF3A23218 | *Z. unicus* | Toepad | Flores Island, Indonesia | -8.5833, 120.5 | 1990-May-08 | WAM | SAMN16986206 | SRS7828873 |
| AMNH110680 | *Z. palpebrosus* | Toepad | Jura, Assam, India | - | 1906-May-02 | AMNH | SAMN16986201 | SRS7828818 |
| AMNH464406 | *Z. palpebrosus* | Toepad | Burnihat, Khasia Hills, India | - | 1949-May-15 | AMNH | SAMN16986213 | SRS7828812 |
| LEIDEN54142 | *Z. simplex* | Toepad | Puli, Taiwan | - | 1968-Jun-21 | Naturalis | SAMN16986204 | SRS7828851 |
| LEIDEN60883 | *Z. melanurus* | Toepad | Cibodas, West Java, Indonesia | - | 1989-Apr-11 | Naturalis | SAMN16986205 | SRS7828862 |
| AMNH801953 | *Z. everetti* | Toepad | Mt. Sugar Loaf, Mindanao Island, Philippines | - | 1969-May-06 | AMNH | SAMN16986203 | SRS7828840 |
| AMNH801954 | *Z. everetti* | Toepad | Mt. Sugar Loaf, Mindanao Island, Philippines | - | 1969-May-12 | AMNH | SAMN16986215 | SRS7828814 |
| AMNH388012 | *Z. flavus* | Toepad | Forest of Mocara Karang, Batavia, Java, Indonesia | - | 1949-Mar-24 | AMNH | SAMN16986216 | SRS7828815 |
| AMNH266601 | *Z. chloris* | Toepad | Djampea Is., between Flores and Celebes, Indonesia | - | 1927-Sep-05 | AMNH | SAMN16986208 | SRS7828895 |
| AMNH700311 | *Z. chloris* | Toepad | Aru, Indonesia | - | 1900-Sep-23 | AMNH | SAMN16986209 | SRS7828811 |
| AMNH700342 | *Z. chloris* | Toepad | Bima, Sumbawa, Indonesia | - | 1896-Aug | AMNH | SAMN16986210 | SRS7828807 |
| AMNH700350 | *Z. chloris* | Toepad | Lombok, Indonesia | - | 1896-May | AMNH | SAMN16986212 | SRS7828809 |
| AMNH700316 | *Z. flavissimus* | Toepad | Tukang Besi, Indonesia | - | 1901-Dec-10 | AMNH | SAMN16986217 | SRS7828816 |
| AMNH700053 | *Z. atricapilla* | Toepad | Kinabalu, Borneo, Malaysia | - | 1896-Oct | AMNH | SAMN16986218 | SRS7828817 |
| AMNH700052 | *Z. atricapilla* | Toepad | Korinchi Peak, Sumatra, Indoensia | - | 1914-May-16 | AMNH | SAMN16986219 | SRS7828819 |
| AMNH700230 | *Z. citrinella* | Toepad | Dammer, Southwest islands, Indonesia | - | 1905-Nov-10 | AMNH | SAMN16986211 | SRS7828808 |
| AMNH700211 | *Z. citrinella* | Toepad | Luang, Southwest islands, Indonesia | - | 1905-Nov-11 | AMNH | SAMN16986207 | SRS7828884 |
| AMNH700247 | *Z. citrinella* | Toepad | Cairncross Island, Australia | - | 1911-Jul-20 | AMNH | SAMN16986220 | SRS7828820 |
| AMNH700180 | *Z. citrinella* | Toepad | Savu, Indonesia | - | 1896-Aug | AMNH | SAMN16986221 | SRS7828821 |
| AMNH347034 | *Z. citrinella* | Toepad | Sumba, Indonesia | - | 1932-Jun-09 | AMNH | SAMN16986222 | SRS7828822 |
| SAMB6173 | *Z. auriventer* | Toepad | Nicobar Island, India | - | 1850s to 1870s | SAMA | SAMN16986239 | SRS7828841 |
| ANWCB30042 | *Z. citrinella* | Toepad | East Timor, Democratic Republic of Timor-Leste | -8.6, 126.3833 | 1973-Apr-13 | ANWC | SAMN16986231 | SRS7828832 |
| NRM572229 | *Z. emiliae* | Toepad | Mt. Poi, Borneo, Malaysia | - | 1923-Nov-03 | NRM | SAMN16986233 | SRS7828834 |
| NRM572230 | *Z. uropygialis* | Toepad | Kai, Indonesia | - | 1897-Dec-16 | NRM | SAMN16986234 | SRS7828835 |
| NRM572232 | *Z. consobrinorum* | Toepad | Lalolei, Southeast Sulawesi, Indonesia | - | 1932-Feb-10 | NRM | SAMN16986235 | SRS7828836 |
| AMNH700399 | *Z. stalkeri* | Toepad | Mansela, Seram, Indonesia | - | 1911-May-30 | AMNH | SAMN16986236 | SRS7828837 |
| AMNH700385 | *Z. buruensis* | Toepad | Waicno, Buru, Indonesia | - | 1912-Jan-18 | AMNH | SAMN16986237 | SRS7828838 |
| AMNH705996 | *Z. novaeguineae* | Toepad | Mt. Hagen, Papua New Guinea | - | 1950-Jul-24 | AMNH | SAMN16986232 | SRS7828833 |
| AMNH338107 | *Z. natalis* | Toepad | Horsburgh Island, Cocos Islands | - | 1939 | AMNH | SAMN16986238 | SRS7828839 |
| AMNH467900 | *Z. atriceps* | Toepad | Maluka Utara, Halmahera, Indonesia | - | 1931-Apr-28 | AMNH | SAMN16986244 | SRS7828846 |
| P062082 | *Z. japonicus* | Toepad | Mt. Katanglad, Mindanao, Philippines | - | 1960-Apr-28 | Peabody | SAMN16986229 | SRS7828830 |
| P097095 | *Z. meyeni* | Toepad | Lumban, Laguna Province, Philippines | - | 1975-Feb-01 | Peabody | SAMN16986230 | SRS7828831 |
| P020253 | *Z. ceylonensis* | Toepad | Nuwara Eliya, Sri Lanka | - | 1950-Aug-31 | Peabody | SAMN16986223 | SRS7828823 |
| P020904 | *Z. japonicus* | Toepad | Mount Fuji, Honshu Island, Japan | - | 1951-Jun-19 | Peabody | SAMN16986224 | SRS7828824 |
| P076595 | *Z. atriceps* | Toepad | Achango, Halmahera, Indonesia | - | 1954-Sep-08 | Peabody | SAMN16986225 | SRS7828825 |
| P076596 | *Z. atriceps* | Toepad | Bacan, Indonesia | - | 1954-Oct-01 | Peabody | SAMN16986226 | SRS7828826 |
| P048642 | *Z. nigrorum* | Toepad | Mt. Simminaplan, Ilocos Norte Province, Philippines | - | 1959-Apr-18 | Peabody | SAMN16986228 | SRS7828828 |
| ZRC34654 | *Z. japonicus* | Toepad | Kerinci, Sumatra, Indonesia | - | Unknown | LKCNHM | SAMN16986240 | SRS7828842 |
| ZRC34613 | *Z. simplex* | Toepad | Kerinci, Sumatra, Indonesia | - | Unknown | LKCNHM | SAMN16986241 | SRS7828843 |
| ZRC90123a | *Z. auriventer* | Toepad | Mt. Poi, Borneo, Malaysia | - | Unknown | LKCNHM | SAMN16986243 | SRS7828845 |
| ZRC34602 | *Z. simplex* | Toepad | Natuna Island, Indonesia | - | Unknown | LKCNHM | SAMN16986245 | SRS7828847 |
| ZRC29329 | *Z. emiliae* | Toepad | Mt. Kinabalu, Borneo, Malaysia | - | Unknown | LKCNHM | SAMN16986242 | SRS7828844 |
| P076601 | *Z. atrifrons* | Toepad | Tomohon, Sulawesi, Indonesia | - | 1954-Oct-28 | Peabody | SAMN16986227 | SRS7828827 |
| NRM572231 | *Z. anomalus* | Toepad | Oeroe, South Sulawesi, Indonesia | - | 1930-Aug-11 | NRM | SAMN16986246 | SRS7828848 |
| OWE010 | *Z. simplex* | Blood | Jurong Bird Park (JBP), Singapore | - | 2016-Jun-24 | JBP donation | SAMN16986278 | SRS7828883 |
| J3590 | *Z. simplex* | Blood | Springleaf, Singapore | 1.4017, 103.8177 | 2015-Jun-26 | Fieldwork | SAMN16986255 | SRS7828858 |
| OWE003 | *Z. simplex* | Blood | Jurong Bird Park, Singapore | - | 2016-Jun-24 | JBP donation | SAMN16986279 | SRS7828885 |
| J3598 | *Z. simplex* | Blood | Springleaf, Singapore | 1.4017, 103.8177 | 2015-Jun-26 | Fieldwork | SAMN16986267 | SRS7828871 |
| KM08 | *Z. melanurus* | Blood | Bogor, Java, Indonesia | - | Unknown | MZB | SAMN16986252 | SRS7828855 |
| KM02 | *Z. melanurus* | Blood | Bogor, Java, Indonesia | - | Unknown | MZB | SAMN16986270 | SRS7828875 |
| KM03 | *Z. melanurus* | Blood | Bogor, Java, Indonesia | - | Unknown | MZB | SAMN16986264 | SRS7828868 |
| WEYE08 | *Z. chloris* | Blood | Bogor, Java, Indonesia | - | Unknown | MZB | SAMN16986271 | SRS7828876 |
| PEN04 | *Z. simplex* | Blood | Kedah, Malaysia | - | 2016 | Fieldwork | SAMN16986253 | SRS7828856 |
| EWE02 | *Z. auriventer* | Blood | Lojing Highlands, Malaysia | 4.5985, 101.4402 | Unknown | Fieldwork | SAMN16986261 | SRS7828865 |
| EWE05 | *Z. auriventer* | Blood | Lojing Highlands, Malaysia | 4.5985, 101.4402 | Unknown | Fieldwork | SAMN16986266 | SRS7828870 |
| EWE07 | *Z. auriventer* | Blood | Lojing Highlands, Malaysia | 4.5985, 101.4402 | Unknown | Fieldwork | SAMN16986281 | SRS7828887 |
| PJG224 | *Z. japonicus* | Tissue | Hawaii, Honolulu, United States | 19.6408, -155.4642 | Unknown | Burke | SAMN16986254 | SRS7828857 |
| NTT004 | *Z. citrinella* | Tissue | Alor, Indonesia | -8.299, 124.740 | 2015-Nov-24 | Fieldwork | SAMN16986259 | SRS7828863 |
| NTT005 | *Z. citrinella* | Tissue | Alor, Indonesia | -8.299, 124.740 | 2015-Nov-24 | Fieldwork | SAMN16986260 | SRS7828864 |
| NTT063 | *Z. citrinella* | Tissue | Kupang, Timor, Indonesia | -10.030, 123.804 | 2015-Nov-27 | Fieldwork | SAMN16986263 | SRS7828867 |
| NTT138 | *Z. citrinella* | Tissue | Mutis, Timor, Indonesia | -9.598, 124.205 | 2015-Dec-01 | Fieldwork | SAMN16986269 | SRS7828874 |
| NTT115 | *Z. citrinella* | Tissue | Rote, Indonesia | -10.787, 123.201 | 2015-Nov-30 | Fieldwork | SAMN16986257 | SRS7828860 |
| NTT123 | *Z. citrinella* | Tissue | Rote, Indonesia | -10.787, 123.201 | 2015-Nov-30 | Fieldwork | SAMN16986251 | SRS7828854 |
| TBU22 | *Z. japonicus* | Tissue | Taliabu, Indonesia | -1.7936, 124.8036 | 2013-Dec-09 | Fieldwork | SAMN16986249 | SRS7828852 |
| TBU23 | *Z. japonicus* | Tissue | Taliabu, Indonesia | -1.7936, 124.8036 | 2013-Dec-09 | Fieldwork | SAMN16986250 | SRS7828853 |
| DOT12552 | *Z. japonicus* | Tissue | Sulawesi Selatan, Indonesia | - | 1998-May-20 | AMNH | SAMN16986248 | SRS7828850 |
| DOT12558 | *Z. chloris* | Tissue | Sulawesi Seletan, Indonesia | - | 1998-May-21 | AMNH | SAMN16986247 | SRS7828849 |
| FL20008 | *Z. simplex* | Tissue | Sichuan, China | 31.0340, 103.1818 | 2014-May-02 | Fieldwork | SAMN16986258 | SRS7828861 |
| FL20009 | *Z. simplex* | Tissue | Sichuan, China | 31.0340, 103.1818 | 2014-May-02 | Fieldwork | SAMN16986280 | SRS7828886 |
| FL20054 | *Z. erythropleurus* | Tissue | Sichuan, China | 31.0300, 103.1818 | 2014-May-15 | Fieldwork | SAMN16986262 | SRS7828866 |
| FL20203 | *Z. erythropleurus* | Tissue | Sichuan, China | 31.0340, 103.1818 | 2014-Jun-02 | Fieldwork | SAMN16986276 | SRS7828881 |
| FL3638 | *Z. palpebrosus* | Tissue | Sichuan, China | 27.2363, 101.5877 | Unknown | Fieldwork | SAMN16986277 | SRS7828882 |
| FL3639 | *Z. palpebrosus* | Tissue | Sichuan, China | 27.2363, 101.5877 | Unknown | Fieldwork | SAMN16986256 | SRS7828859 |
| B56431 | *Z. luteus* | Tissue | King River Road, Western Australia | -15.5768, 128.1409 | 2014-Sep-11 | ANWC | SAMN16986265 | SRS7828869 |
| B56434 | *Z. luteus* | Tissue | King River Road, Western Australia | -15.5768, 128.1409 | 2014-Sep-11 | ANWC | SAMN16986275 | SRS7828880 |
| B60155 | *Z. lateralis* | Tissue | Western Australia | -31.677, 128.883 | 2017-Aug-06 | ANWC | SAMN16986268 | SRS7828872 |
| B52557 | *Z. fuscicapilla* | Tissue | Muller Range, Papua New Guinea | -5.6566, 142.3046 | 2009-Sep-16 | ANWC | SAMN16986272 | SRS7828877 |
| DOT19901 | *Z. hypoxanthus* | Tissue | Bismarck, Papua New Guinea | -3.2749, 151.9304 | 2012-Oct-16 | AMNH | SAMN16986274 | SRS7828879 |
| RCKB2303 | *Z. atrifrons* | Tissue | Dako, South Sulawesi, Indonesia | - | 2018-Jul-11 | MVZ | SAMN16986283 | SRS7828889 |
| RCKB2264 | *Z. atrifrons* | Tissue | Dako, South Sulawesi, Indonesia | - | 2018-Jul-05 | MVZ | SAMN16986282 | SRS7828888 |
| Ac01 | *Z. melanurus* | Blood | Banda Aceh, Sumatra, Indonesia | - | 2018-Dec-07 | Fieldwork | SAMN16986284 | SRS7828890 |
| Ac04 | *Z. melanurus* | Blood | Banda Aceh, Sumatra, Indonesia | - | 2018-Dec-07 | Fieldwork | SAMN16986285 | SRS7828891 |
| Ac05 | *Z. melanurus* | Blood | Banda Aceh, Sumatra, Indonesia | - | 2018-Dec-07 | Fieldwork | SAMN16986286 | SRS7828892 |
| ENG1 | *Z. simplex* | Feather | Enggano, Sumatra, Indonesia | - | Unknown | Fieldwork | SAMN16986273 | SRS7828878 |
| Afr01 | *Z. senegalensis* | Tissue | Africa | - | Unknown | Captive | SAMN16986287 | SRS7828893 |
| BG02 | *Z. chloris* | Blood | Bogor, Java, Indonesia | - | Unknown | MZB | SAMN16986288 | SRS7828894 |
| BG06 | *Z. chloris* | Blood | Bogor, Java, Indonesia | - | Unknown | MZB | SAMN16986289 | SRS7828896 |
| LMB66* | *Z. chloris* | Tissue | Kai Kecil, Indonesia | - | 2014-Jul-14 | MVZ |  |  |
| ALS937* | *Z. chloris* | Tissue | Kur, Indonesia | - | Aug-14 | MVZ |  |  |
| LMB30* | *Z. citrinella* | Tissue | Wetar, Indonesia | - | 2013-Jul-19 | MVZ |  |  |
| LMB79* | *Z. citrinella* | Tissue | Babar, Indonesia | - | 2014-Jul-27 | MVZ |  |  |
| LMB8* | *Z. citrinella* | Tissue | Timor, Indonesia | - | 2013-Jul-9 | MVZ |  |  |
| LMB99* | *Z. citrinella* | Tissue | Tanimbar, Indonesia | - | 2014-Jul-30 | MVZ |  |  |
| A22083* | *Z. chloris* | Tissue | Sumbawa, Indonesia | -8.225, 119.0056 | 1988-Oct-22 | WAM |  |  |
| Z5848* | *Z. luteus* | Tissue | Cape Range National Park, Western Australia, Australia | -21.97, 114.47 | 1996-Oct-11 | NMV |  |  |
| Z50534* | *Z. natalis* | Tissue | Christmas Island, Australia | -10.5, 105.67 | Unknown | NMV |  |  |
