# Supplementary material for: Phylogenomics of white-eyes, a ‘great speciator’, reveals Indonesian archipelago as the center of lineage diversity
Source: eLife. 2020 Dec 22;9:e62765. doi: 10.7554/eLife.62765 (PMC7775107; doi:10.7554/eLife.62765)
Supplement: Supplementary file 2. — All species were assigned to their respective ranges of occurrence (see Figure 1): Afrotropical, West Indian Ocean (WIO), Asia, Philippines, Indonesian Archipelago (Indo), Melanesian Archipelago (Mel), Micronesian Archipelago (Mic), Australia and/or others. [file elife-62765-supp2.docx]

Supplementary file 2. A list of species assignable to one of the three main *Zosterops* clades (Indo-African, Asiatic, Australasian), either on the basis of our study (shaded in grey) or based on previous studies which has a bootstrap support of at least 90. All recognized species in the present study were assigned to the respective range of occurrence (see Fig. 1): Afrotropical, West Indian Ocean (WIO), Asia, Philippines, Indonesian Archipelago (Indo), Melanesian Archipelago (Mel), Micronesian Archipelago (Mic), Australia and/ or others.

| **No.** | **Species** | **Clade affinity** | **Evidence** | **Region** | **Remarks** |
| --- | --- | --- | --- | --- | --- |
| 1 | *Z. palpebrosus* | Indo-African | Our study | Asia |  |
| 2 | *Z. ceylonensis* | Indo-African | Our study | Asia |  |
| 3 | *Z. mouroniensis* | Indo-African | Cai et al. (2019) | WIO |  |
| 4 | *Z. semiflavus* | Indo-African | Cai et al. (2019) | WIO |  |
| 5 | *Z. olivaceus* | Indo-African | Cai et al. (2019) | WIO |  |
| 6 | *Z. chloronothos* | Indo-African | Cai et al. (2019) | WIO |  |
| 7 | *Z. mauritianus* | Indo-African | Cai et al. (2019) | WIO |  |
| 8 | *Z. borbonicus* | Indo-African | Our study | WIO |  |
| 9 | *Z. melanocephalus* | Indo-African | Cai et al. (2019) | Afrotropical |  |
| 10 | *Z. brunneus* | Indo-African | Cai et al. (2019) | Afrotropical |  |
| 11 | *Z. poliogastrus* | Indo-African | Cai et al. (2019), Warren et al. (2006) | Afrotropical |  |
| 12 | *Z. kikuyuensis* | Indo-African | Cai et al. (2019) | Afrotropical |  |
| 13 | *Z. ficedulinus* | Indo-African | Cai et al. (2019) | Afrotropical |  |
| 14 | *Z. griseovirescens* | Indo-African | Cai et al. (2019) | Afrotropical |  |
| 15 | *Z. feae* | Indo-African | Cai et al. (2019) | Afrotropical |  |
| 16 | *Z. lugubris* | Indo-African | Cai et al. (2019) | Afrotropical |  |
| 17 | *Z. leucophaeus* | Indo-African | Cai et al. (2019) | Afrotropical |  |
| 18 | *Z. silvanus* | Indo-African | Cai et al. (2019) | Afrotropical |  |
| 19 | *Z. senegalensis* | Indo-African | Our study | Afrotropical |  |
| 20 | *Z. capensis* | Indo-African | Cai et al. (2019) | Afrotropical |  |
| 21 | *Z. pallidus* | Indo-African | Our study | Afrotropical |  |
| 22 | *Z. vaughani* | Indo-African | Cai et al. (2019) | Afrotropical |  |
| 23 | *Z. modestus* | Indo-African | Cai et al. (2019) | WIO |  |
| 24 | *Z. maderaspatanus* | Indo-African | Cai et al. (2019), Warren et al. (2006) | WIO |  |
| 25 | *Z. mayottensis* | Indo-African | Cai et al. (2019), Warren et al. (2006) | WIO |  |
| 26 | *Z. kirki* | Indo-African | Cai et al. (2019), Warren et al. (2006) | WIO |  |
| 27 | *Z. abyssinicus* | Indo-African | Cox et al. (2014) | Afrotropical |  |
| 28 | *Z. virens* | Indo-African | Oatley et al. (2012), Moyle et al. (2009) | Afrotropical |  |
| 29 | *Z. flavus* | Indo-African | Our study | Indo |  |
| 30 | *Z. nigrorum* | Asiatic | Our study | Philippines |  |
| 31 | *Z. atricapilla* | Asiatic | Our study | Indo |  |
| 32 | *Z. erythropleurus* | Asiatic | Our study | Asia |  |
| 33 | *Z. japonicus* | Asiatic | Our study | Asia/ Philippines /Indo |  |
| 34 | *Z. simplex* | Asiatic | Our study | Asia/ Indo |  |
| 35 | *Z. everetti* | Asiatic | Our study | Philippines |  |
| 36 | *Z. auriventer* | Asiatic | Our study | Asia/ Indo |  |
| 37 | *Z. meyeni* | Asiatic | Our study | Philippines | lumped with *Z. japonicus* (based on this study) |
| 38 | *Z. emiliae* | Australasian | Our study | Indo |  |
| 39 | *Z. natalis* | Australasian | Our study | Others: Indian Ocean |  |
| 40 | *Z. chloris* | Australasian | Our study | Indo | consists of two species (based on this study) |
| 41 | *Z. citrinella* | Australasian | Our study | Indo |  |
| 42 | *Z. melanurus* | Australasian | Our study | Indo |  |
| 43 | *Z. unicus* | Australasian | Our study | Indo |  |
| 44 | *Z. flavissimus* | Australasian | Our study | Indo |  |
| 45 | *Z. luteus* | Australasian | Our study | Australia |  |
| 46 | *Z. consobrinorum* | Australasian | Our study | Indo |  |
| 47 | *Z. uropygialis* | Australasian | Our study | Indo |  |
| 48 | *Z. anomalus* | Australasian | Our study | Indo |  |
| 49 | *Z. atriceps* | Australasian | Our study | Indo |  |
| 50 | *Z. atrifrons* | Australasian | Our study | Indo |  |
| 51 | *Z. stalkeri* | Australasian | Our study | Indo |  |
| 52 | *Z. buruensis* | Australasian | Our study | Indo |  |
| 53 | *Z. hypoxanthus* | Australasian | Our study | Mel |  |
| 54 | *Z. fuscicapilla* | Australasian | Our study | Mel |  |
| 55 | *Z. novaeguineae* | Australasian | Our study | Mel |  |
| 56 | *Z. lateralis* | Australasian | Our study | Australia/ Mel |  |
| 57 | *Z. flavifrons* | Australasian | Cai et al. (2019) | Mel |  |
| 58 | *Z. superciliosus* | Australasian | Cai et al. (2019), Moyle et al. (2009) | Mel |  |
| 59 | *Z. gibbsi* | Australasian | Cai et al. (2019) | Mel |  |
| 60 | *Z. lacertosus* | Australasian | Cai et al. (2019) | Mel |  |
| 61 | *Z. minutus* | Australasian | Cornetti et al. (2015) | Mel |  |
| 62 | *Zosterops undescribed. (Meratus White-eye)* | Australasian | Shakya et al. (2018) | Indo |  |
| 63 | *Zosterops undescribed (Wangi-wangi White-eye)* | Australasian | O’Connell et al. (2019) | Indo |  |
| 64 | *Z. rotensis* | N.A. | N.A. | Mic |  |
| 65 | *Z. saypani* | N.A. | N.A. | Mic |  |
| 66 | *Z. semperi* | N.A. | N.A. | Mic |  |
| 67 | *Z. hypolais* | N.A. | N.A. | Mic |  |
| 68 | *Z. finschii* | N.A. | N.A. | Mic |  |
| 69 | *Z. ponapensis* | N.A. | N.A. | Mic |  |
| 70 | *Z. cinereus* | N.A. | N.A. | Mic |  |
| 71 | *Z. oleagineus* | N.A. | N.A. | Mic |  |
| 72 | *Z. chrysolaemus* | N.A. | N.A. | Mel |  |
| 73 | *Z. minor* | N.A. | N.A. | Mel |  |
| 74 | *Z. meeki* | N.A. | N.A. | Mel |  |
| 75 | *Z. mysorensis* | N.A. | N.A. | Mel |  |
| 76 | *Z. crookshanki* | N.A. | N.A. | Mel |  |
| 77 | *Z. metcalfii* | N.A. | N.A. | Mel |  |
| 78 | *Z. stresemanni* | N.A. | N.A. | Mel |  |
| 79 | *Z. hamlini* | N.A. | N.A. | Mel |  |
| 80 | *Z. rendovae* | N.A. | N.A. | Mel |  |
| 81 | *Z. vellalavella* | N.A. | N.A. | Mel |  |
| 82 | *Z. sanctaecrucis* | N.A. | N.A. | Mel |  |
| 83 | *Z. samoensis* | N.A. | N.A. | Mel |  |
| 84 | *Z. explorator* | N.A. | N.A. | Mel |  |
| 85 | *Z. xanthochroa* | N.A. | N.A. | Mel |  |
| 86 | *Z. luteirostris* | N.A. | N.A. | Mel |  |
| 87 | *Z. splendidus* | N.A. | N.A. | Mel |  |
| 88 | *Z. kulambangrae* | N.A. | N.A. | Mel |  |
| 89 | *Z. albogularis* | N.A. | N.A. | Mel |  |
| 90 | *Z. tenuirostris* | N.A. | N.A. | Mel |  |
| 91 | *Z. inornatus* | N.A. | N.A. | Mel |  |
| 92 | *Z. rennellianus* | N.A. | N.A. | Mel |  |
| 93 | *Z. griseotinctus* | N.A. | N.A. | Mel |  |
| 94 | *Z. murphyi* | N.A. | N.A. | Mel |  |
| 95 | *Z. strenuus* | N.A. | N.A. | Others: Tasman Sea |  |
| 96 | *Z. grayi* | N.A. | N.A. | Indo |  |
| 97 | *Z. somadikartai* | N.A. | N.A. | Indo |  |
| 98 | *Z. nehrkorni* | N.A. | N.A. | Indo |  |
| 99 | *Z. kuehni* | N.A. | N.A. | Indo |  |
| 100 | *Z. flavilateralis* | N.A. | N.A. | Afrotropical |  |
| 101 | *Z. eurycricotus* | N.A. | N.A. | Afrotropical |  |
| 102 | *Z. socotranus* | N.A. | N.A. | Afrotropical | split from *Z. abyssinicus* (Martins et al. 2020) |
| 103 | *Zosterops* undescribed (Somalia) | N.A. | N.A. | Afrotropical |  |
| 104 | *Z. anderssoni* | N.A. | N.A. | Afrotropical | split from *Z. senegalensis* (Martins et al. 2020) |
| 105 | *Z. jacksoni* | N.A. | N.A. | Afrotropical |  |
| 106 | *Z. quanzae* | N.A. | N.A. | Afrotropical |  |
| 107 | *Z. stuhlmanni* | N.A. | N.A. | Afrotropical |  |
| 108 | *Z. stenocricotus* | N.A. | N.A. | Afrotropical |  |
|  | *Z. kulalensis* | N.A. | N.A. | Afrotropical | lumped with *Z. poliogastrus* (Martins et al. 2020) |
|  | *Z. kaffensis* | N.A. | N.A. | Afrotropical |  |
